# Potential links between brown adipose tissue, circadian dysregulation, and suicide risk

**DOI:** 10.3389/fnins.2023.1196029

**Published:** 2023-06-09

**Authors:** Jan Sarlon, Timo Partonen, Undine E. Lang

**Affiliations:** ^1^University Psychiatric Clinics (UPK), University of Basel, Basel, Switzerland; ^2^Department of Public Health and Welfare, Finnish Institute for Health and Welfare (THL), Helsinki, Finland

**Keywords:** brown adipose fat tissue, suicide risk, circadian disruption, clozapine, body mass index

## Abstract

Circadian desynchronizations are associated with psychiatric disorders as well as with higher suicidal risk. Brown adipose tissue (BAT) is important in the regulation of body temperature and contributes to the homeostasis of the metabolic, cardiovascular, skeletal muscle or central nervous system. BAT is under neuronal, hormonal and immune control and secrets batokines: i.e., autocrine, paracrine and endocrine active substances. Moreover, BAT is involved in circadian system. Light, ambient temperature as well as exogen substances interact with BAT. Thus, a dysregulation of BAT can indirectly worsen psychiatric conditions and the risk of suicide, as one of previously suggested explanations for the seasonality of suicide rate. Furthermore, overactivation of BAT is associated with lower body weight and lower level of blood lipids. Reduced body mass index (BMI) or decrease in BMI respectively, as well as lower triglyceride concentrations were found to correlate with higher risk of suicide, however the findings are inconclusive. Hyperactivation or dysregulation of BAT in relation to the circadian system as a possible common factor is discussed. Interestingly, substances with proven efficacy in reducing suicidal risk, like clozapine or lithium, interact with BAT. The effects of clozapine on fat tissue are stronger and might differ qualitatively from other antipsychotics; however, the significance remains unclear. We suggest that BAT is involved in the brain/environment homeostasis and deserves attention from a psychiatric point of view. Better understanding of circadian disruptions and its mechanisms can contribute to personalized diagnostic and therapy as well as better assessment of suicide risk.

## Introduction

Although brown adipose tissue (BAT) was discovered in mammals already in 1551 as well as in humans in 1902, the first systematic description of its distribution in humans was proven with autopsies in 1972 (Heaton, 1972). For a long time, BAT was believed to have no physiological relevance in adult humans (Nedergaard et al., 2007; Cypess et al., 2009; Virtanen et al., 2009). However, different research groups proved the contrary (Nedergaard et al., 2007; Cypess et al., 2009; Virtanen et al., 2009; van Marken Lichtenbelt et al., 2009). Recently, BAT has been attracting increasing interest of researchers (Saito et al., 2020) through different fields of biological and medical sciences including cell and molecular biology (Chen and Pfeifer, 2017; Claflin et al., 2022), endocrinology (Villarroya et al., 2017; Sentis et al., 2021), metabolic science (Alcalá et al., 2019; Yang and Stanford, 2022; Ziqubu et al., 2023) and neurosciences (Partonen, 2015; Ortiz-Alvarez et al., 2022; Sideromenos et al., 2022b).

Brown adipose tissue plays a key role in adaptive non-shivering thermogenesis and resting energy expenditure (Emmett et al., 2017; Chondronikola and Sidossis, 2019). Moreover, it is involved in metabolic homeostasis, regulating body weight, glucose and lipid metabolism (Rui, 2017; Sustarsic et al., 2018). In addition, recent research has shown that BAT act as an endocrine organ and interacts with the central nervous system (Villarroya et al., 2017; Martins et al., 2022; Till et al., 2022). BAT is also involved in circadian system (Yang et al., 2011; Meng et al., 2023), and its overactivation might contribute to the manifestation of mood disorders (Partonen, 2015), be a potential endophenotype of depression (Partonen, 2013) and contribute to a higher suicide risk (Holopainen et al., 2014).

In the following, the physiology of BAT as well as interactions between BAT, circadian clock, exogen factors like seasonality or drugs, and their impact on the risk of suicide will be reviewed. Furthermore, a potential link between body mass index (BMI), BAT dysregulation and suicide risk will be briefly discussed.

## Physiology of human BAT

Both white and brown adipose tissue contribute to the regulation of food intake and energy turnover. While white adipose tissue stores energy, brown adipose tissue produces heat. By that, BAT contributes to the maintenance of body temperature during cold exposure and to the elevated core temperature during several behavioral states, including wakefulness, the acute phase response (fever), and stress (Morrison and Madden, 2014).

Fat cells (adipocytes) in BAT contain a high density of mitochondria with a specific protein, called uncoupling protein 1 (UCP1), which uncouples oxidation from ATP synthesis and converts glucose and fat acids into heat (Chen et al., 2022). BAT is under neuronal, hormonal and immune control (Rui, 2017; Sentis et al., 2021; Till et al., 2022). Furthermore, nutritive aspects can modulate its function. While capsaicin, caffeine (Van Schaik et al., 2022), rutin (Cheng et al., 2022) or unsaturated long chain fatty acids (Fosch et al., 2023) stimulate the BAT thermogenesis, food additives or pesticides (Wang et al., 2021) as well as saturated fat (Fosch et al., 2023) can inhibit the thermogenesis in BAT.

The predominant neural pathway and main trigger of BAT thermogenesis is the sympathetic pathway via beta-2 adrenergic receptor in human BAT (Straat et al., 2023), in rodents via β3-adrenergic receptor signaling (Cero et al., 2021). Stress leads to an increase thermogenesis in BAT via sympathetic activation. Neurons in dorsomedial hypothalamus receive a stress-related excitation from the dorsal peduncular cortex and dorsal tenia tecta in the ventromedial prefrontal cortex (Blessing et al., 2006; Nakamura and Morrison, 2022). Furthermore, BAT is modulated by ventromedial hypothalamus as well as by orexin, a neuropeptide produced in lateral hypothalamus (Martins et al., 2016). Orexin plays an important role in feeding and arousal as well as regulates also body temperature and energy homeostasis (Madden et al., 2012). Orexin deficiency in narcolepsy type 1 is associated with obesity due to impaired BAT thermogenesis, despite reduced food intake (Sellayah et al., 2011).

BAT secrets signaling molecules, called batokines, including peptides, metabolites, lipids, or microRNAs (Yang and Stanford, 2022) with autocrine, paracrine or endocrine function. To this group of batokines belong among others Interleukin 6, Myostatin, Neuregulin 4, Fibroblast Growth Factor 21 or Vascular Endothelial Growth Factor A (Martins et al., 2022). BAT can be thus considered as one of stress-responsive endocrine organs (Qing et al., 2020).

## BAT and circadian system

Physiological and behavior functions are rhythmic, regulated via endogenous pacemakers—circadian clocks. Wake and sleep cycles, body (core) temperature, hormone secretion, food intake or metabolic processes belong to the main circadian (about 24 h) rhythm. The central pacemaker (“central clock”) is located in the hypothalamic suprachiasmatic nucleus (SCN). The molecular mechanism of the circadian clock in mammals is generated by a cell-autonomous transcriptional autoregulatory feedback loop. The core clock genes include “clock circadian regulator,” aka circadian locomotor output cycles kaput (Clock), its analog “neuronal pas domain protein 2” (Npas2) and “basic helix–loop–helix ARNT like 1, aka brain-and-muscle ARNT-like protein 1” (Bmal1)—which encode activators; as well as period circadian regulators (Per1, Per2, Per3) and cryptochrome circadian regulators (Cry1 and Cry2)—which encode repressors (Takahashi, 2017; Bolsius et al., 2021).

The central clocks orchestrate all other clocks in the organism. All tissues, including WAT and BAT show circadian rhythmicity, which means, they have their own “clocks.” The circadian axis hypothalamus-adipose tissue emphasizes the importance of functional adipose tissue clock for the circadian regulation of energy homeostasis (Heyde et al., 2021) and its disruption can affect metabolic processes (Dollet and Zierath, 2019). Furthermore, circadian rhythm of core temperature can modulate the amplitude of circadian gene expression (Goh et al., 2021). Expression of core clock genes in brown fat, but not in white fat, is highly responsive to cold exposure (Li et al., 2013). Cryptochromes react not only to light exposure and ultraviolet radiation, but also to geomagnetic activity, and they take part in the energy balance involving BAT in mammals (López et al., 2010; Foley et al., 2011; Partonen, 2015).

## BAT and BMI

BAT is associated with lower BMI and lower visceral obesity (Takeda et al., 2023) and regulates glucose metabolism and insulin sensitivity (Stanford et al., 2013). This is a rationale for the view that BAT can be considered a potential target to treat obesity and its comorbidities (Martins et al., 2022).

In obese individuals, BAT mass and activity are minimized due to reduced cell proliferation and preadipocyte differentiation and increased apoptosis (Alcalá et al., 2019). Changes in BAT (hypertrophy and whitening, disturbances of batokines) are described also in diabetes type two in rodents (Ziqubu et al., 2023).

## Suicidality and BMI

In a prospective study of over 45,000 men, the suicide mortality rate was strongly inversely related to BMI, even when adjusted for medical illness, dietary factors, antidepressant use, physical activity, and social support (Mukamal et al., 2007).

A retrospective analysis on 387 people who died by suicide demonstrated a significant association between BMI decrease within the last 12 month and increased risk for suicide after adjustment for demographic characteristics, mental disorders, and comorbidities (Hecht et al., 2021). Furthermore, suicide attempters with mood disorders exhibit less adiposity, expressed as a reduced BMI, waist circumference and serum triglycerides, when compared to non-attempters (da Graça Cantarelli et al., 2015).

However, the findings are inconsistent Overweight/obesity was significant associated with the risk of suicidal ideation and suicide attempts among adolescents in low and middle income countries (Zhang et al., 2022). Furthermore, some authors point out that body weight has a complex relationship with physical and mental health including suicidal thoughts and behavior, which may not be possible to accurately capture with a singular metric such as BMI (Harris et al., 2022).

## Suicidality and seasonality

Studies from both the Northern and the Southern hemisphere in both mid-latitude and sub-tropical climate type report a seasonal pattern for suicides with a late spring/summer peak in suicide rates (Christodoulou et al., 2012; Dixon and Kalkstein, 2018). A review of 45 studies from 26 different countries confirmed those patterns (Galvão et al., 2018). Moreover, the seasonal variation is stronger for violent suicide (Christodoulou et al., 2012). Bioclimatic factors (sunlight exposure and global sunlight radiation, ambient temperature, precipitations) are considered to play a role in the seasonality of suicide (Christodoulou et al., 2012).

There is solid evidence of association between ambient temperature and its change and suicidality. Marion et al. showed that a combination of cooler preceding 3 months together with a warmer current month (both with a difference of 2.5°C) was associated with an increase of 30% in elderly suicide rate (Marion et al., 1999). Furthermore, there is a significant correlation between suicidality and rapidly increasing temperature (Volpe et al., 2008). This finding was further corroborated by analysis, showing that warm temperatures, especially abnormally warm temperatures during cool months, increased the risk for suicide (Kaufman and Fitzmaurice, 2021). This is congruent with earlier findings from a large population based study analyzing more than 250,000 deaths by suicide (Holopainen et al., 2014). Two peaks within a year could be found one main in May, the second one, less pronounced, in October. In the studied latitude, temperature changes from April to May as well as the temperature drop from September to October coincided with the seasonal suicide maximums in May and October. The authors could show that in May, the temperature difference between current and precedent month was the highest within the year, in line with the highest suicide risk (Holopainen et al., 2014).

In another study, large changes in solar insolation between winter and summer were associated with an increased risk of suicide attempts in bipolar 1 disorder (Bauer et al., 2021). Further, suicide rate was higher on days after a short-term increase in air temperature on the day before as well as on days with a lower cloud cover (higher insolation) in analysis focusing on both cloud cover and temperature changes (Schneider et al., 2020). More daylight due to natural conditions like summer at high latitude does seem to play a subordinate role, as there was only minimal increases in suicide rate (by 0.75%) for each extra hour of daylight increase within Norwegian regions (Hernæs and Skyrud, 2022).

## Circadian disruption, BAT dysregulation, and suicidal behavior

### Circadian disruption

Increasing clinical and preclinical evidence supports significant crosstalk between circadian clock and psychiatric disorders (Singla et al., 2022) and especially for mood disorders (Lavebratt et al., 2010; Kovanen et al., 2013; Bunney et al., 2015; Mendoza, 2019; Sirignano et al., 2022).

Chronobiological alterations were associated with passive and active suicidal ideation or preparation (Palagini et al., 2021) and with suicide attempts (Grant et al., 2022). Circadian rhythm disturbances are a common symptom among individuals with mood disorders (Vadnie and McClung, 2017) and can be a mood disorder phenotype (Carpenter et al., 2021). Findings of involvement of circadian system in rapid antidepressant response (ketamine and sleep deprivation) support the bidirectional relationship between circadian system and mood disorders (Sato et al., 2022; Singla et al., 2022).

### BAT dysregulation

BAT thermogenesis plays a significant role in the overall maintenance of the circadian rhythm of core temperature (Yang et al., 2011). There is evidence suggesting that the evolutionarily ancient temperature resetting response is a universal “zeitgeber” and enhances the internal circadian synchronization (Buhr et al., 2010). Thermogenesis in BAT is suppressed by light exposure (Meng et al., 2023), and a prolonged day length decreases sympathetic input into BAT (Kooijman et al., 2015). Earlier, it has been hypothesized that an overactivity of BAT with a compromised heat tolerance in spring (sudden temperature changes and more daylight after a cold period) can trigger anxiety and psychomotor agitation, and thus increase suicidal risk (Holopainen et al., 2014).

Disturbances in the circadian system and over-activated BAT can underlie some key features of a depression endophenotype, such as blunted circadian amplitudes with elevated core temperature in the night, loss of weight among others (Partonen, 2013). It remains unclear, if and to which extent the sudden inhibitory signals (more daylight, higher temperatures) to previously activated BAT (due to cold periods or psychiatric symptoms) leads to circadian mismatch or disruption (see also later in text).

## Effects of clozapine and lithium on BAT

The inhibition of brown adipogenesis by atypical or second-generation antipsychotics (SGA) is believed to be one possible mechanism to explain weight gain induced by SGA (Oh et al., 2012; Kristóf et al., 2016). Some first-generation antipsychotics like chlorpromazine can also reduce emotional hyperthermia, while non-dopaminerg mechanism of action has been suggested (Blessing et al., 2017). Weight gain and inhibition of BAT are common for SGA’s. However, clozapine shows the greatest effect among all SGA’s. In mouse brown adipocytes, both clozapine and quetiapine downregulated the brown adipogenesis, quetiapine being weaker than clozapine, whereas ziprasidone did not alter the differentiation. Clozapine also significantly inhibited the mRNA expression of lipogenic genes as well as adiponectin (Oh et al., 2012). In patients with schizophrenia, the treatment with SGA aripiprazole or clozapine was associated with a decreased BAT activity (measured by plasma levels of bone morphogenetic protein 8b, a batokine secreted by BAT), as compared to drug-free patients, and the decrease was more significant for clozapine compared to aripiprazole (Chen et al., 2022).

There is evidence suggesting also specific effects of clozapine on BAT, compared to other SGA’s. Expression of UPC1 was elevated by clozapine, but not with six other antipsychotics in human adipocytes *ex vivo*, leading to an induction of beige cells rather than the classical brown phenotype (Kristóf et al., 2016). The relevance of this finding remains unclear. However, clozapine relevantly reduces stress-induced thermogenesis in BAT and might be effective in life-threatening hyperthermia induced by 3,4-methylenedioxymethamphetamine (MDMA, aka ecstasy) in humans (Blessing et al., 2006). Another proposed mechanism is a blockage of thermogenic effects of orexin by clozapine (Monda et al., 2004).

There are only few studies concerning the effects of lithium on adipocytes. There is evidence that lithium can downregulate the brown adipocyte differentiation (Rodríguez de la Concepción et al., 2005). However, low-dose lithium chloride leads to an inhibition of glycogen-synthase kinase and thus to a beiging-like effect in WAT obtained from mice (Geromella et al., 2022). This finding can be compared to a similar effect of clozapine, i.e., induction of beige cells from white adipocytes (Kristóf et al., 2016).

Summarizing the above presented findings, we present following hypothesis that would need to be confirmed by experimental studies:

- BAT dysregulation contributes to—and can be seen as a marker of—circadian disruption.
- Circadian disturbance (that means also BAT dysregulation) can exacerbate psychiatric disorders and aggravate suicidal behavior.
- BAT is actively involved in crosstalk between brain/circadian system, periphery organs and environment.
- Normalization of the circadian system can reduce suicidal risk, while different components can be targeted.

### BAT overactivity or dysregulation?

Compromised heat tolerance due to previously overactivated BAT (recent cold period, psychological stress) could explain findings, which indicate higher suicidal risk with increased ambient temperature, especially if such increase is sudden. One possible mechanism behind that might be enhanced agitation or anxiety, as previously suggested (Holopainen et al., 2014; Partonen, 2015). The fact, that solar insolation can increase suicidal risk, would strengthen the hypothesis of circadian disruption and dysregulation of BAT (upregulated BAT, sudden increase in the inhibitory input in contrast with previous activation, since both light exposure and higher temperatures inhibit BAT thermogenesis), rather than a simple overactivation as hypothesized earlier (Partonen, 2012) and tested against the key circadian clock gene Cry2 (Sokolowska et al., 2021). It seems more likely that pathways can adapt within weeks rather than within days.

It remains unclear, however, which mechanisms are responsible for this effect, and several possible pathways can be assumed. For example, these may consist of mismatch within the circadian clock, increased circadian disruption, endocrine effects of BAT (batokines), or some other feedback-loop between BAT, circadian system and behavior.

As some authors proposed (Papadopoulos et al., 2005), increased daylight can first induce changes in motivational or action components than improvement of mood. That would be in line with the observation on nearly 70,000 confirmed suicides in Austria between 1970 and 2010, where the hours of sunshine up to 10 days prior to suicide positively correlated with suicide rates, whereas more daily sunshine 14–60 days previously was associated with lower rates of suicide (Vyssoki et al., 2014).

Rather than increased activity of BAT alone, more a dysregulation between BAT and other circadian components might be crucial. Otherwise, simply cold exposure would present a risk factor from the psychiatric perspective, but there is no evidence for that. We hypothesize that sudden changes (in ambient temperature, insolation, etc.) can cause an imbalance of slow adaptation processes, and might aggravate circadian disturbance.

As mentioned above, there are findings supporting a correlation between a weight loss and a higher suicide rate. If we regard this phenomenon from the perspective of the herein proposed BAT dysregulation theory, weight loss can be considered as a clinical symptom of overactivated BAT. On the other hand, an overactivated BAT can be one possible reason for body weight loss in patients with major psychiatric disorder. In line with Hecht et al. (2021), we assume that the causality between weight loss and suicide mortality cannot be ascertained: the weight change may either directly increase suicidal risk or be an indicator of mental health symptoms (Hecht et al., 2021). Loss of appetite, delusions, or motivational problems with acquisition or preparing of food as caused by symptom severity in major psychiatric disorders (major depression, schizophrenia) would also contribute to weight loss. However, presented findings demonstrated that there is an increased risk for suicide after BMI reduction even after adjustment for mental disorders. Further, an altered adipose tissue-brain communication may play a role here (da Graça Cantarelli et al., 2015). Since BAT activation accelerates plasma clearance of triglycerides as a result of increased uptake into BAT (Bartelt et al., 2011), reduced serum triglycerides in suicide attempters might refer to hyperactivated BAT. Therefore, reduction of BMI can be hypothesized as an independent risk factor. Following question is rising—can weight gain under therapy represent a “protective” biomarker regarding risk of suicide?

Furthermore, we speculate that BAT dysregulation could be one possible common denominator which links compromised heat tolerance, reduced BMI and blood lipids. However, the exact role of BAT here remains to be elucidated, since the BAT dysregulation might be only a proxy of the primary cause (such as circadian disruption). An assessment of BAT activity in individuals on higher suicide risk (for example with present suicide thoughts or plans) before and after treatment would yield worthwhile data.

### Crosstalk between BAT, brain, and periphery organs

Diverse crosstalks between BAT, brain and other organs have been described (Contreras et al., 2015; Moreno-Navarrete and Fernandez-Real, 2019; Qing et al., 2020; Ortiz-Alvarez et al., 2022; Till et al., 2022).

**Figure 1:**
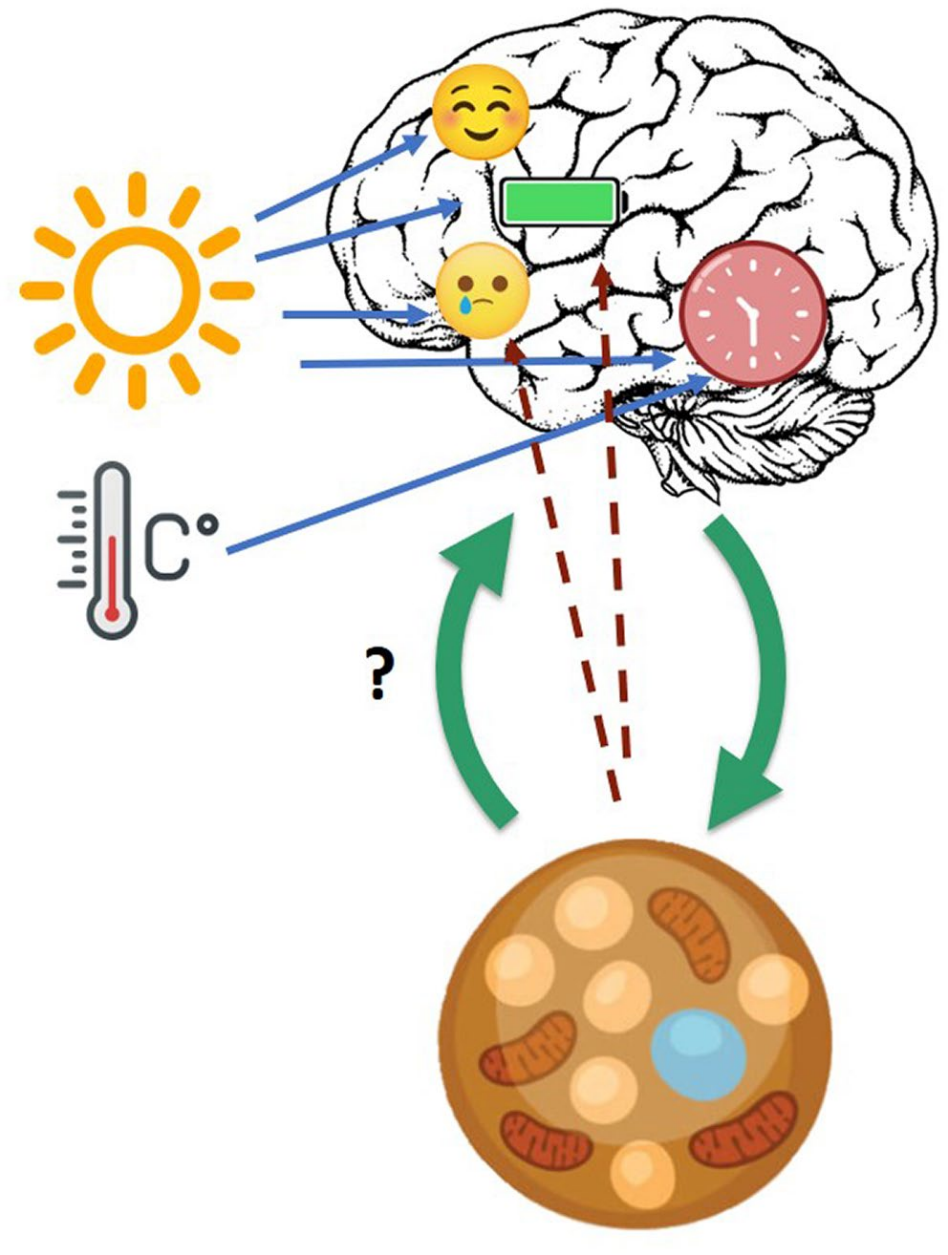
Cross-talk between brain, circadian clock, and BAT. Blue arrows: daylight and ambient temperature affect mood, energy level, and circadian clock. Green arrows: cross-talk between brain and brown adipose tissue in general. Dotted line arrows: hypothesized influence of BAT on mood and energy level.

In the following some evidence suggesting possible pathways of interaction between BAT and brain is presented, especially the “bottom-up” one (the “top-down” pathway is well described), as presented in Figure 1.

### Batokines

As presented above, BAT secrets signaling proteins with paracrine and endocrine functions. Interleukin 6 (IL-6) is produced by different cells, among others in BAT. Interleukin-6 is a cytokine not only involved in inflammation and infection responses, but also in the regulation of metabolic, regenerative, and neural processes (Scheller et al., 2011). IL-6 is known as a pro-inflammatory cytokine, however it has also anti-inflammatory effects (Mauer et al., 2014). IL-6 is involved in the physiological homeostasis of neural tissue and in neuropsychiatric disorders (Rothaug et al., 2016; Khandaker et al., 2018). Furthermore, there is evidence that a higher serum level of IL-6 is associated with suicide behavior (González-Castro et al., 2021; Castillo-Avila et al., 2022).

### Expression of UPC-1

Uncoupling protein 1 is responsible for thermogenesis in the mitochondria of BAT. Since UPC-1 expression was detected also in other tissues, including the hypothalamus (Claflin et al., 2022), it could be another possible link between brain and BAT. In mice, UCP-1 modulated anxiety-like behavior in a temperature-dependent manner (Sideromenos et al., 2022a).

### Sensory inflow from BAT

Two research groups demonstrated neuronal loop from brain to BAT and back via sensory afferents from BAT to several brain regions, mainly the hypothalamus, raphe pallidus, periaqueductal gray, olivary areas, parabrachial nuclei, raphe nuclei, and reticular areas (Vaughan and Bartness, 2012; Ryu et al., 2015).

## BAT biomarkers

The best way to measure volume and activity of BAT is the use of 18 [F]-Fluorodeoxyglucose (18-F-FDG) in combination with a positron emission tomography/computed tomography (PET/CT). Second, as periphery biomarkers, UPC-1, or BAT-derived exomes can be used (Giralt et al., 2016; Chen and Pfeifer, 2017; Alito et al., 2022). Batokines have already been discussed above, however, they are not BAT-specific. Third, the assessment of the supraclavicular skin temperature using thermal sensors as promising non-invasive measures (e.g., iButtons) was related to cold-induced 18-F-FDG in women (Martinez-Tellez et al., 2019). However, it is not established yet as an alternative to 18-F-FDG yet.

## Conclusion and future perspectives

In summary, we suggest that BAT pathophysiology plays a potential role in psychiatric conditions and deserves more attention in neurosciences. The recent study on beta-adrenergic profile in human BAT compared to rodents (Straat et al., 2023) pointed out the need for more studies in human population.

**Figure 2:**
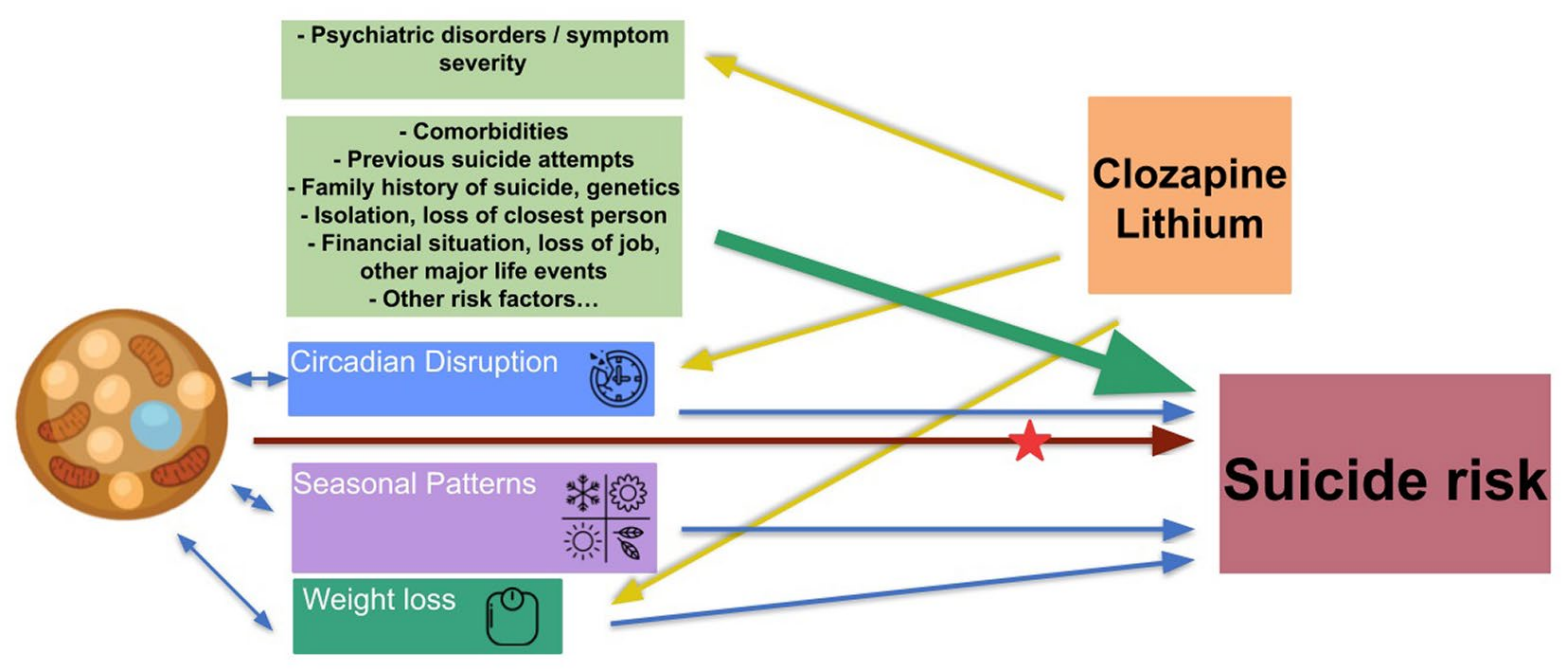
Suicide risk factors, BAT, clozapine, and lithium. Green arrows: known risk factors of suicide. Blue arrows: risk factors involving BAT. Yellow arrows: known effects/targets of clozapine and lithium. Brown arrows: hypothetical pathway leading from BAT dysregulation to increase suicide risk, to be elucidate. Red star: unclear if and how clozapine and lithium affect those pathways.

Understanding which combination of environmental and endogenous factors, and under which conditions increase suicide risk, may contribute to a better prevention of suicide. Figure 2 presents an overview of suicide risk factors, including those where the involvement of BAT is likely or assumed as well as the role of lithium and clozapine. Whether or not BAT activity is elevated in individuals with suicidal ideation and behavior, remains to be seen in experimental studies. One way to find this out would be to measure BAT activity (direct or indirect assessment). The time spent in the natural ambient temperature can vary, not only between regions and cultures, but also between individuals and might be associated with life standards. Since humans actively regulate their ambient temperature, mid-term assessment of BAT activity together with the monitoring of the (individual) ambient temperature would be one possible experimental design. Thus, one potential study design could implement measurements of individual ambient temperature when assessing the impact of meteorological conditions or seasonal patterns of mood or behavior. Monitoring changes in BAT activity under therapy with rapid acting antidepressants, lithium, or clozapine would be an example of one plausible study design to address clinical aspects.

## Data availability statement

The original contributions presented in the study are included in the article/supplementary material, further inquiries can be directed to the corresponding author.

## Author contributions

JS, TP, and UL contributed to conception of the manuscript. JS wrote the first draft of the manuscript, supervised by UL. TP contributed to literature and conclusions and reviewed all figures. All authors substantially contributed to the manuscript revision, read and approved the submitted version of the manuscript.

## Conflict of interest

The authors declare that the research was conducted in the absence of any commercial or financial relationships that could be construed as a potential conflict of interest.

## Publisher’s note

All claims expressed in this article are solely those of the authors and do not necessarily represent those of their affiliated organizations, or those of the publisher, the editors and the reviewers. Any product that may be evaluated in this article, or claim that may be made by its manufacturer, is not guaranteed or endorsed by the publisher.
